# Comparison of Performance between ARC-HBR Criteria and PRECISE-DAPT Score in Patients Undergoing Percutaneous Coronary Intervention

**DOI:** 10.3390/jcm10122566

**Published:** 2021-06-10

**Authors:** Sun Young Choi, Moo-Hyun Kim, Kwang-Min Lee, Yeo-Gyeong Ko, Chan-Ho Yoon, Min-Kyeong Jo, Sung-Cheol Yun

**Affiliations:** 1 Department of Cardiology, Dong-A University Hospital, Busan 49201, Korea; kmu5041@hanmail.net (S.Y.C.); tnt849@hanmail.net (K.-M.L.); choiko9120@naver.com (Y.-G.K.); chyoon1113@naver.com (C.-H.Y.); rudrudrud123@gmail.com (M.-K.J.); 2 Department of Biomedical Laboratory Science, Daegu Health College, Daegu 41453, Korea; 3 Department of Clinical Epidemiology and Biostatistics, University of Ulsan College of Medicine, Asan Medical Center, Seoul 05505, Korea; ysch97@amc.seoul.kr

**Keywords:** bleeding, risk, score, percutaneous coronary intervention

## Abstract

The proper management of bleeding risk in patients undergoing percutaneous coronary intervention (PCI) is critical. Recently, the Academic Research Consortium for High Bleeding Risk (ARC-HBR) criteria have been proposed as a standardized tool for predicting bleeding risk. We sought to compare the predictive performance of ARC-HBR criteria and the PRECISE-DAPT score for bleeding in Korean patients undergoing PCI. We recruited 1418 consecutive patients undergoing PCI from January 2012 through December 2018 (Dong-A University Medical Center, Busan, Korea). The ARC-HBR and PRECISE-DAPT scores showed a high AUC for three bleeding definitions (AUC 0.75 and 0.77 for BARC 3 to 5; AUC 0.68 and 0.71 for TIMI minor to major; AUC 0.81 and 0.82 for GUSTO moderate to severe, respectively) and all-cause death (AUC 0.82 and 0.82, respectively). When compared with the ARC-HBR score, the discriminant ability of the PRECISE-DAPT score was not significantly different for bleeding events and all-cause death. The ARC-HBR criteria and PRECISE-DAPT scores demonstrated reasonably good discriminatory capacity with respect to 1-year bleeding events in Korean patients treated with DAPT, regardless of the bleeding definition. Our findings also suggest that the simple PRECISE-DAPT score is as useful as ARC-HBR criteria in predicting bleeding and all-cause death after PCI.

## 1. Introduction

Dual antiplatelet therapy (DAPT) with aspirin and a P2Y12 inhibitor after percutaneous coronary intervention (PCI) reduces ischemic events. However, prolonged DAPT increases bleeding risk, which has been associated with critical adverse events [1,2,3,4]. For this reason, the proper identification and management of patients with high bleeding risk (HBR) is critically important, and several tools have been investigated in randomized trials to predict bleeding risk [5,6,7,8]. However, standardization of a tool for this purpose has not been achieved to date, due to differences in the characteristics of the derivation cohorts, the timing after PCI, and genetic differences associating with ethnicity.

In 2017, a simple five-item risk score called the Predicting Bleeding Complication in Patients Undergoing Stent Implantation and Subsequent Dual Antiplatelet Therapy (PRECISE-DAPT) score was created as a standardized tool for patients treated with DAPT after PCI in a large, pooled dataset of contemporary, randomized clinical trials, implementing different DAPT duration strategies [9]. Recently, the Academic Research Consortium for HBR (ARC-HBR) criteria have been proposed to standardize the definition of HBR in patients undergoing PCI, based on consensus from an expert panel [10,11,12]. To date, assessments of the performance of the ARC-HBR criteria in clinical practice have been limited. We sought to measure the performance of the ARC-HBR criteria in Asian patients undergoing PCI and to draw a comparison with the PRECISE-DAPT score.

## 2. Materials and Methods

### 2.1. Study Population

Between January 2012 and December 2018, a total of 1418 post-PCI patients (Dong-A University Medical Center, Busan, Republic of Korea) receiving maintenance DAPT were recruited for our prospective observational cross-sectional study. Written informed consent was obtained from all patients, and the study protocol was approved by the Ethical Review Board of Dong-A University Hospital. We excluded patients with active bleeding or those who had undergone major surgery within the prior 4 weeks, and those receiving treatment with oral anticoagulants.

### 2.2. Definition

The PRECISE-DAPT scores and ARC-HBR criteria were assessed using the patients’ clinical characteristics. The PRECISE-DAPT score was determined using an online calculator with five variables (age, creatinine clearance, hemoglobin, white blood cell count and previous spontaneous bleeding) [9]. The individual rating for variables, established for each score, was assigned. The total scores for each patient were calculated by summing the individual result for each prognostic variable included in the score. Patients were classified as belonging to the HBR group if at least 1 major or 2 minor ARC-HBR criteria were met, and patients with 1 minor criterion were classified as no-HBR [10]. The ARC-HBR criteria are as follows: major criteria such as oral anticoagulation, severe chronic kidney disease (estimated glomerular filtration [eGFR] < 30 mL/min), thrombocytopenia (platelet count < 100 × 10^9^/L), severe anemia (hemoglobin < 11 g/dL), liver cirrhosis, active malignancy within 12 months and/or ongoing requirement for treatment (excluding nonmelanoma skin cancer) or prior hemorrhagic stroke, and minor criteria such as age ≥ 75 years, moderate chronic kidney disease (eGFR 30–59 mL/min), mild anemia (hemoglobin 11–12.9 g/dL for men and 11–11.9 g/dL for women) or prior ischemic stroke. Additionally, the ARC-HBR criteria required modification for the ARC-HBR scores, which can be compared with the PRECISE-DAPT score [13]. The ARC-HBR score was calculated by adding 1 point for any major criterion and 0.5 points for any minor criterion.

### 2.3. Clinical Endpoint

The primary endpoint for the analysis was all-cause death and bleeding complications defined according to three different bleeding severity scales: Bleeding Academic Research Consortium (BARC) 3 to 5 bleeding [14], Thrombolysis in Myocardial Infarction (TIMI) minor or major [15] and Global Use of Strategies To Open coronary arteries (GUSTO) moderate or severe [16].

### 2.4. Statistical Analysis

Continuous variables are expressed as mean values with standard deviations, and categorical variables are presented as frequencies (percentages). Comparisons between two mean values for continuous variables were analyzed by Student’s t-test. Categorical variables were compared with Pearson’s chi-square or Fisher’s exact test. The PRECISE-DAPT scores were stratified into three risk categories of bleeding (low, moderate and high). The predictive values of the PRECISE-DAPT and ARC-HBR scores were assessed using a Cox regression model and receiver operating characteristics (ROC) curve analysis (using MedCalc Version 12.2.1, MedCalc software, Mariakerke, Belgium) [17]. The prognostic utility of the risk models for major bleeding was assessed by deriving their C-statistics, using ROC curves. In general, a model with a C-statistic above 0.70 is considered to have acceptable discriminatory capacity [18]. The C-statistics for the three risk models were compared to each other using a nonparametric test [19]. The calibration of the models was evaluated using the Hosmer–Lemeshow goodness-of-fit statistical analysis. Net reclassification improvement (NRI) represents the average weighted improvement in discrimination, while integrated discrimination improvement (IDI) takes into account the change in the estimated prediction probabilities as a continuous variable and represents the average improvement in predictive probability. The impact of the reclassification procedure using the superior score was assessed using the NRI approach. Positive values of NRI indicate the predominance for correct reclassification, while negative values indicate a predominance of incorrect reclassification. *p*-values < 0.05 were considered to indicate significance. Statistical analyses were performed using SPSS Version 18.0 (SPSS Inc., Chicago, IL, USA).

## 3. Results

### 3.1. Baseline Characteristics

The study cohort comprised 1418 patients who were treated with DAPT. The baseline demographics and clinical characteristics are shown in Table 1. Of the total patients, 502 patients (35.4%) were classified as belonging to the HBR group and 916 patients (64.6%) in the no-HBR group. Compared to their no-HBR counterparts, HBR patients were older (aged ≥ 75 years: 54%) and more frequently female (39.2%). HBR was also associated with diabetes mellitus, hypertension, current smoking and prior stroke history, and differences in renal function, anemia, platelet count, liver function, and cancer.

### 3.2. Clinical Outcome Risks Associated with HBR

**Figure 1:**
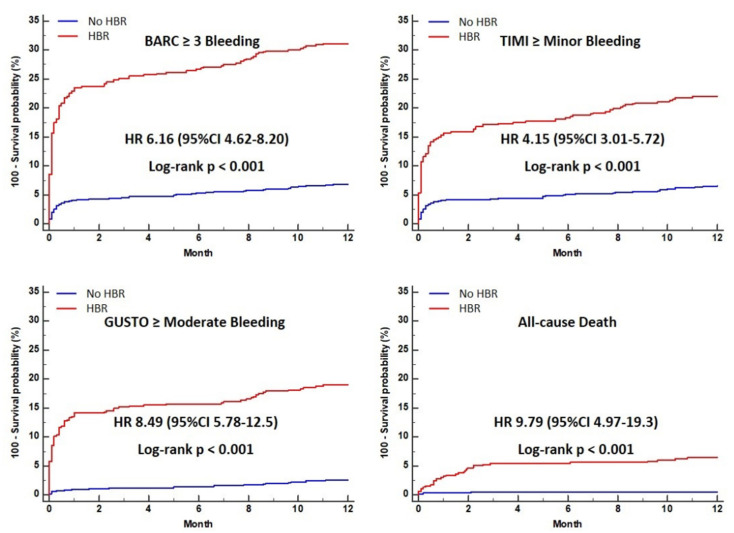
Cumulative incidence curve of clinical outcomes in HBR and no-HBR groups. BARC, Bleeding Academic Research Consortium; CI, confidence interval; GUSTO, Global Use of Strategies To Open coronary arteries; HBR, high bleeding risk; HR, hazard ration; TIMI, Thrombolysis in Myocardial Infarction.

The cumulative incidence of 1-year clinical events, according to HBR, is shown in Table 2. The incidence of BARC 3 or 5 bleeding, TIMI minor or major bleeding, and GUSTO moderate or severe bleeding, were significantly higher in the HBR group than in the no-HBR group (31.1% vs. 6.9%, 21.9% vs. 6.6%, and 18.9% vs. 2.5%, *p* < 0.001, respectively). The cumulative incidence of all-cause death was significantly higher in patients with HBR, compared to those without HBR (Table 2). HBR patients had a significantly higher risk of BARC 3 or 5 bleeding (hazard ration [HR] 5.21, 95% confidence interval [CI] 4.62–8.20, *p* < 0.001), TIMI minor or major bleeding (HR 4.15, 95% CI 3.01–5.72, *p* < 0.001), and GUSTO moderate to severe bleeding (HR 8.49, 95% CI 5.78–12.5, *p* < 0.001), than no-HBR patients. HBR was also associated with higher risk of all-cause death (HR 9.79, 95% CI 4.97–19.3, *p* < 0.001) compared to the no-HBR group (Table 2 and Figure 1).

### 3.3. Effect of Individual ARC-HBR Criteria and ARC-HBR Scores on Clinical Outcomes

The risk of ARC-HBR major and minor criteria is summarized in Table 3. Severe anemia was associated with the highest risk for BARC 3 to 5 (HR 4.37, 95% CI 3.34–5.72, *p* < 0.001) and GUSTO moderate to severe bleeding (HR 9.17, 95% CI 6.33–13.3, *p* < 0.001). The factor associated with the highest risk was active malignancy for TIMI minor to major bleeding (HR 2.72, 95 % CI 1.21–6.16) and all-cause death (HR 13.0, 95% CI 5.06–33.4, *p* < 0.001). However, among the ARC-HBR major criteria, oral anticoagulation was not associated with the risk of three bleeding events (BARC 3 to 5, TIMI minor to major, and GUSTO moderate to severe) and all-cause death, while minor criteria such as old age and moderate chronic kidney disease increased the risk of three bleeding definitions and all-cause death by more than two-fold.

**Figure 2:**
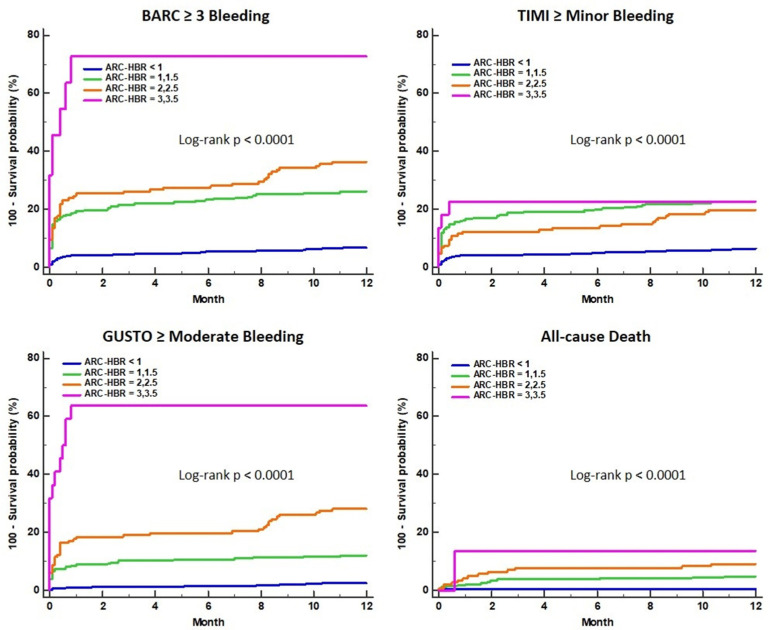
Cumulative incidence curve of clinical outcomes for different ARC-HBR scores. ARC-HBR, Academic Research Consortium for High Bleeding Risk criteria; BARC, Bleeding Academic Research Consortium; GUSTO, Global Use of Strategies To Open coronary arteries; TIMI, Thrombolysis in Myocardial Infarction.

The predictive performance of the ARC-HBR score for 1-year clinical outcomes was greatest for higher scores compared to lower scores (Table 4). The relative risk of three bleeding events and all-cause death continuously increased for the higher scores compared with ARC-HBR scores of <1. The cumulative incidence curve shows that patients with higher ARC-HBR scores had a higher risk of adverse clinical outcomes (Figure 2).

### 3.4. Comparison of Predictive Performance of ARC-HBR and PRECISE-DAPT Scores

**Figure 3:**
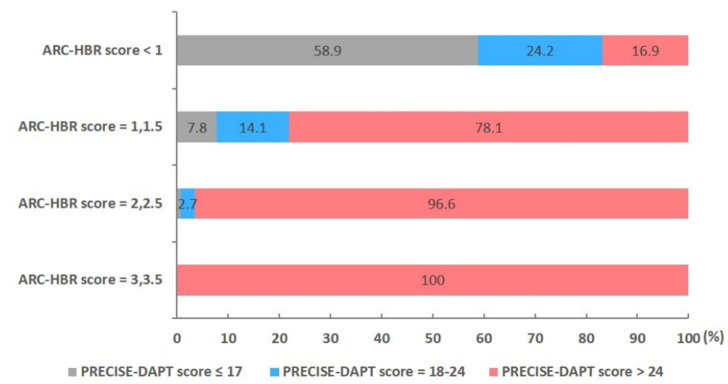
Distribution of risk categories for the PRECISE-DAPT score according to different ARC-HBR scores. ARC-HBR, Academic Research Consortium for High Bleeding Risk criteria; PRECISE-DAPT, Predicting Bleeding Complication in Patients Undergoing Stent Implantation and Subsequent Dual Antiplatelet therapy.

The distribution of the risk categories in the PRECISE-DAPT score according to the different ARC-HBR scores is presented in Figure 3. In total, 58.9% patients with an ARC-HBR score of 0 or 0.5 (no-HBR group) met the threshold for the low-risk category of the PRECISE-DAPT score (≤17). The higher ARC-HBR scores enabled broader inclusion in the high-risk category of the PRECISE-DAPT score (>24). Meanwhile, 78.1% of patients with ARC-HBR scores of 1 (one major or two minor criteria) or 1.5 (one major and one minor criteria, or three minor criteria) and most patients (>95%) with ARC-HBR scores of ≥2 satisfied the high PRECISE-DAPT score criteria. Table 5 presents the discriminatory capacity of the ARC-HBR and PRECISE-DAPT scores in predicting clinical outcomes at one year, including assessing the area under the curve (AUC). In the C-statistics analysis, the ARC-HBR and PRECISE-DAPT scores showed high AUC values for three bleeding definitions (AUC 0.75 and 0.77 for BARC 3 to 5; AUC 0.68 and 0.71 for TIMI minor to major; AUC 0.81 and 0.82 for GUSTO moderate to severe, respectively) and all-cause death (AUC 0.82 and 0.82, respectively). When compared with the ARC-HBR score, the discriminant ability of the PRECISE-DAPT score was not significantly different for predicting three bleeding definitions and all-cause death. When compared with the PRECISE-DAPT, the ARC-HBR was not significantly superior in terms of net reclassification improvement (NRI) or integrated discrimination improvement (IDI) (Table 6).

## 4. Discussion

We found that the ARC-HBR criteria demonstrated reasonable discriminatory capacity with respect to 1-year bleeding in Korean patients treated with DAPT, regardless of the bleeding definition (BARC 3 to 5, TIMI minor to major, and GUSTO moderate to severe). In addition, the discriminatory ability of the PRECISE-DAPT score, a simple five-item prediction algorithm for bleeding, was similar to the ARC-HBR criteria, a recently developed consensus risk stratification tool. Furthermore, 78.1% patients with ARC-HBR scores of 1 or 1.5 and most patients (>95%) with ARC-HBR scores of ≥2 were stratified into the high PRECISE-DAPT score category. Patients with HBR also carried a six-fold risk of BARC 3 to 5 bleeding, a four-fold increase in TIMI minor to major bleeding, an eight-fold risk of GUSTO moderate to severe bleeding, and a nine-fold higher risk of all-cause death compared with no-HBR patients. An increased number of ARC-HBR criteria being fulfilled was also associated with an incrementally higher incidence of bleeding events and all-cause death.

Major bleeding is one of the most common serious adverse events in acute coronary syndrome [20]. In this clinical setting, there is a strong relationship between bleeding and mortality, with major bleeding associated with a 60% increase in risk of hospital death [21]. Potent DAPT can increase the number of patients at high risk of bleeding complications. Simple standardized risk stratification is therefore needed for clinical decision-making, relating to the intensity and duration of DAPT after PCI, via an accurate bleeding risk assessment.

Recently, ARC-HBR has been proposed as a consensus-based definition for HBR in patients undergoing PCI and comprises 12 clinical criteria identified as major and minor, supported by published evidence [10]. Meanwhile, the PRECISE-DAPT score is a simple prediction algorithm with five risk factors (age, creatinine clearance, hemoglobin, white-blood-cell count, and previous spontaneous bleeding) as a standardized tool for the prediction of 1-year bleeding during DAPT [9]. An additional advantage of PRECISE-DAPT is that it was derived from eight multicenter randomized clinical trials with independent adjudication of the events. The 2017 European Society of Cardiology Focused Update on DAPT recommended the PRECISE-DAPT score for guidance over the duration of DAPT [22]. In the previous study, we validated the performance of this score for predicting bleeding events in Korean patients undergoing PCI [23]. The simplified PRECISE-DAPT score without WBC count has a similar predictive value for bleeding after PCI [24].

To date, investigations of the performance of the ARC-HBR criteria and the PRECISE-DAPT score in real clinical practice have been limited, and so we modified the ARC-HBR criteria to an ARC-HBR score to compare the predictive ability of these two scoring methods. For the C-statistics analysis, the discriminatory ability of PRECISE-DAPT was not inferior to the ARC-HBR criteria in complications. Moreover, more than 95% of patients are considered to be in the high PRECISE-DAPT category if at least two major or four minor criteria are met. We believe the strength of these findings is supported by the incorporation of various bleeding definitions (BARC, TIMI and GUSTO criteria), which capture the different bleeding complications.

### Study Limitations

We note several limitations to our study design. As a single-center retrospective study, the results provided are hypothesis-generating, and the scores evaluated have not yet been validated in an all-comer population, being designed primarily to predict events within one year after the index procedure. It should also be emphasized that there may be confounders to our analysis that could impact the conclusions, including potentially missed bleeding events. Among the ARC-HBR major criteria, oral anticoagulation was not associated with the risk of three bleeding definitions (BARC 3 to 5, TIMI minor to major, and GUSTO moderate to severe) or all-cause death, because a small percentage of patients were included in this study. Furthermore, we did not evaluate ischemic risk. Ischemia and bleeding share overlapping risk factors (e.g., older age, renal dysfunction) and ACS patients have a higher risk of death/ischemia and bleeding complications.

## 5. Conclusions

The ARC-HBR criteria and PRECISE-DAPT risk scores demonstrated reasonably good discriminatory capacity with respect to 1-year bleeding in Korean patients treated with DAPT, regardless of the bleeding definition. Our findings suggest that the relatively simple PRECISE-DAPT score is as accurate as ARC-HBR criteria in predicting bleeding and all-cause death after PCI. Most HBR patients defined by the ARC-HBR criteria were stratified into the high-risk category of the PRECISE-DAPT score.

## Figures and Tables

**Table 1:** Baseline characteristics.

Variable	Overall (*n* = 11,418)	HBR Group (*n* = 502)	No-HBR Group (*n* = 916)	*p*-Value ^a^
Age, year	66.2 ± 24.3	73.2 ± 9.3	62.3 ± 9.5	<0.001
Age ≥ years	346 (24.4)	271 (54.0)	75 (8.2)	<0.001
Female gender	395 (27.9)	197 (39.2)	198 (21.6)	<0.001
BMI, kg/m^2^	24.3 ± 3.2	23.3 ± 3.2	24.8 ± 3.0	<0.001
Diabetes mellitus	613 (43.2)	274 (54.6)	339 (37.0)	<0.001
Hypertension	916 (64.6)	378 (75.3)	538 (58.7)	<0.001
Dyslipidemia	659 (46.5)	233 (46.6)	462 (50.4)	0.141
Current smoking	368 (26.0)	67 (13.3)	301 (32.9)	<0.001
Diagnosis				<0.001
Angina	855 (60.3)	261 (52.0)	594 (64.8)	
NSTEMI	449 (31.7)	211 (42.0)	238 (26.0)	
STEMI	114 (8.0)	30 (6.0)	84 (9.2)	
Previous myocardial infarction	354 (25.0)	139 (27.7)	215 (23.5)	0.100
Previous hemorrhagic stroke	17 (1.2)	17 (1.3)	0 (0)	<0.001
Previous ischemic stroke	131 (9.2)	94 (18.7)	37 (4.0)	<0.001
Previous antiplatelet therapy	554 (50.5)	213 (57.1)	341 (47.0)	0.002
Previous oral anticoagulation	8 (0.6)	8 (1.6)	0 (0)	<0.001
Moderate CKD (eGFR 30–59)	246 (17.3)	198 (39.4)	48 (5.2)	<0.001
Severe CKD (eGFR < 30)	85 (6.0)	85 (16.9)	0 (0)	<0.001
Mild anemia (Hb 11–12.9 g/dL for male, 11–11.9 g/dL for female)	346 (24.4)	178 (35.5)	168 (18.3)	<0.001
Severe anemia (Hb < 11 g/dL)	236 (16.6)	236 (47.0)	0 (0)	<0.001
Platelet < 100 × 10^9^/L	22 (1.6)	22 (4.4)	0 (0)	<0.001
Liver cirrhosis	27 (1.9)	27 (5.4)	0 (0)	<0.001
Malignancy	19 (1.3)	19 (3.8)	0 (0)	<0.001
Medication				<0.001
Clopidogrel	1208 (85.2)	453 (90.2)	755 (82.4)	
New P2Y12 inhibitor	180 (12.7)	38 (7.6)	142 (15.5)	

Values are *n* (%) or mean ± standard deviation. ^a^ Between HBR and no-HBR group. BMI, body mass index; CKD, chronic kidney disease; eGFR, estimated glomerular filtration rate; Hb, hemoglobin; HBR, high bleeding risk; NSTEMI, non-ST elevation myocardial infarction; STEMI, ST elevation myocardial infarction.

**Table 2:** Event rates in the HBR group.

Event	HBR Group No. of Events (*n* = 502)	No-HBR Group No. of Events (*n* = 916)	*p*-Value	HR (95%CI)	*p*-Value
BARC ≥ 3 bleeding (*n* = 219)	156 (31.1%)	63 (6.9%)	<0.001	6.12 (4.62–8.20)	<0.001
TIMI ≥ minor bleeding (*n* = 170)	110 (21.9%)	60 (6.6%)	<0.001	4.15 (3.01–5.72)	<0.001
GUSTO ≥ mod bleeding (*n* = 118)	95 (18.9%)	23 (2.5%)	<0.001	8.49 (5.78–12.5)	<0.001
All-cause death (*n* = 37)	32 (6.4%)	5 (0.5%)	<0.001	9.79 (4.97–19.3)	<0.001

Values are *n* (%) compared to the no-HBR group as the reference. BARC, Bleeding Academic Research Consortium; CI, confidence interval; GUSTO, Global Use of Strategies To Open coronary arteries; HBR, high bleeding risk; HR, hazard ratio; TIMI, Thrombolysis in Myocardial Infarction.

**Table 3:** Risk of clinical events according to Academic Research Consortium for High Bleeding Risk (ARC-HBR) criteria.

Variables		BARC > 3a No. of Events (*n* = 219)	HR (95% CI)	*p*-Value	TIMI ≥ Minor No. of Events (*n* = 170)	HR (95% CI)	*p*-Value	GUSTO ≥ Mod No. of Events (*n* = 118)	HR (95% CI)	*p*-Value	Death No. of Events (*n* = 37)	HR (95% CI)	*p*-Value
Minor criteria	Age ≥ 75	93 (26.9%)	2.49 (1.90–3.26)	<0.001	67 (19.4%)	2.13 (1.56–2.89)	<0.001	53 (15.3%)	2.66 (1.85–3.83)	<0.001	23 (6.6%)	5.24 (2.70–10.2)	<0.001
	Moderate CKD	67 (27.2%)	2.28 (1.71–3.05)	<0.001	52 (21.1%)	2.24 (1.62–3.11)	<0.001	37 (15.0%)	2.27 (1.54–3.35)	<0.001	17 (6.9%)	4.18 (2.19–7.98)	<0.001
	Mild Anemia	61 (17.6%)	1.22 (0.91–1.64)	0.198	57 (16.5%)	1.62 (1.18–2.23)	0.003	21 (6.1%)	0.66 (0.42–1.06)	0.084	10 (2.9%)	1.15 (0.56–2.37)	0.712
	Prior IS	34 (26.0%)	1.93 (1.34–2.79)	<0.001	23 (13.5%)	1.58 (1.02–2.45)	0.042	15 (11.5%)	1.48 (0.86–2.55)	0.156	5 (3.8%)	1.55 (0.60–3.97)	0.364
Major criteria	Severe CKD	39 (45.9%)	4.06 (2.86–5.74)	<0.001	19 (22.4%)	2.05 (1.27–3.31)	0.003	32 (37.6%)	7.00 (4.66–10.5)	<0.001	8 (9.4%)	4.38 (2.00–9.59)	<0.001
	Severe Anemia	92 (39.0%)	4.37 (3.34–5.72)	<0.001	50 (21.2%)	2.26 (1.63–3.14)	<0.001	72 (30.5%)	9.17 (6.33–13.3)	<0.001	18 (7.6%)	4.90 (2.57–9.34)	<0.001
	Thrombocytopenia	8 (36.4%)	2.75 (1.36–5.57)	0.005	3 (13.6%)	1.16 (0.37–3.62)	0.804	8 (36.4%)	5.51 (2.68–11.3)	<0.001	3 (13.6%)	5.89 (1.81–19.2)	0.003
	Prior ICH	5 (29.4%)	2.11 (0.87–5.12)	0.099	2 (11.8%)	0.92 (0.24–3.87)	0.962	4 (23.5%)	3.15 (1.16–8.53)	0.024	0 (0%)	-	-
	Liver cirrhosis	9 (33.3%)	2.50 (1.28–4.88)	0.007	6 (22.2%)	2.04 (0.90–4.61)	0.086	7 (25.9%)	3.65 (1.70–7.83)	0.001	2 (7.4%)	3.08 (0.74–12.8)	0.122
	Active malignancy	10 (52.6%)	3.78 (2.01–7.14)	<0.001	6 (31.6%)	2.72 (1.21–6.16)	0.016	8 (42.1%)	5.95 (2.90–12.2)	<0.001	5 (26.3%)	13.0 (5.06–33.4)	<0.001
	OAC	1 (12.5%)	0.83 (1.12–5.93)	0.850	1 (12.5%)	1.10 (0.15–7.83)	0.928	0 (0%)	-	-	0 (0%)	-	-

Values are *n* (%). BARC, Bleeding Academic Research Consortium; CI, confidence interval; CKD, chronic kidney disease; GUSTO, Global Use of Strategies To Open coronary arteries; HR, hazard ration; ICH, intracranial hemorrhage; IS, ischemic stroke; OAC, oral anticoagulation; TIMI, Thrombolysis in Myocardial Infarction.

**Table 4:** Risk of clinical events according to Academic Research Consortium for High Bleeding Risk (ARC-HBR) score.

Variable	BARC ≥ 3 (*n* = 219)		TIMI ≥ Minor (*n* = 170)		GUSTO ≥ Moderate (*n* = 118)		All-Cause Death (*n* = 37)	
ARC-HBR score < 1 (*n* = 916)	63 (6.9%)		60 (6.6%)		23 (2.5%)		5 (0.6%)	
ARC-HBR score = 1,1.5 (*n* = 334)	87 (26.1%)		76 (22.8%)		40 (12.0%)		16 (4.8%)	
ARC-HBR score = 2,2.5 (*n* = 146)	53 (36.3%)		29 (19.9%)		41 (28.1%)		13 (9.1%)	
ARC-HBR score = 3,3.5 (*n* = 22)	16 (72.7%)		5 (22.7%)		14 (63.6%)		3 (13.6%)	
	**BARC ≥ 3**		**TIMI ≥ Minor**		**GUSTO ≥ Moderate**		**All-Cause Death**	
	**HR (95%CI)**	***p*-Value**	**HR (95%CI)**	***p*-Value**	**HR (95%CI)**	***p*-Value**	**HR (95%CI)**	***p*-Value**
ARC-HBR score < 1	Reference	<0.001	Reference	<0.001	Reference	<0.001	Reference	<0.001
ARC-HBR score = 1,1.5	4.24 (3.07–5.87)		3.82 (2.64–5.52)		5.04 (3.26–7.81)		8.91 (4.14–19.4)	
ARC-HBR score = 2,2.5	6.13 (3.86–9.75)		3.22 (1.93–5.37)		12.6 (6.73–23.6)		17.1 (5.70–51.3)	
ARC-HBR score = 3,3.5	18.1 (4.60–70.9)		4.00 (1.10–14.5)		40.8 (6.59–252.4)		26.7 (1.81–395.5)	

Values are *n* (%). BARC, Bleeding Academic Research Consortium; CI, confidence interval; GUSTO, Global Use of Strategies To Open coronary arteries; HBR, high bleeding risk; HR, hazard ration; TIMI, Thrombolysis in Myocardial Infarction.

**Table 5:** Discriminative power of the ARC-HBR score and PRECISE-DAPT score for predicting 1-year bleeding according to definition.

	ARC-HBR Score		PRECISE-DAPT Score	
	AUC (95% CI)	*p*-Value	AUC (95% CI)	*p*-Value
BARC ≥ 3 bleeding	0.75 (0.73–0.78)	<0.001	0.77 (0.75–0.80)	<0.001
TIMI ≥ minor bleeding	0.68 (0.66–0.71)	<0.001	0.71 (0.68–0.73)	<0.001
GUSTO ≥ moderate bleeding	0.81 (0.79–0.83)	<0.001	0.82 (0.80–0.84)	<0.001
All-cause death	0.82 (0.80–0.84)	<0.001	0.82 (0.80–0.84)	<0.001
	**z Statistics (95% CI)**		***p*-Value**	
BARC ≥ 3 bleeding	1.589 (−0.005–0.047)		0.112	
TIMI ≥ minor bleeding	1.530 (−0.007–0.053)		0.126	
GUSTO ≥ moderate bleeding	0.816 (−0.019–0.472)		0.414	
All-cause death	0.178 (−0.049–0.058)		0.859	

ARC-HBR, Academic Research Consortium for High Bleeding Risk criteria; AUC, area under the curve; BARC, Bleeding Academic Research Consortium; CI, confidence interval; GUSTO, Global Use of Strategies To Open coronary arteries; PRECISE-DAPT, Predicting Bleeding Complication in Patients Undergoing Stent Implantation and Subsequent Dual Antiplatelet Therapy; TIMI, Thrombolysis in Myocardial Infarction.

**Table 6:** Net reclassification improvement and integrated discriminatory improvement for 1-year bleeding.

Comparison	Event	Bleeding Correctly Reclassified, P (n1)	No Bleeding Correctly Reclassified, P (n2)	NRI	*p*	IDI	*p*
PRECISE-DAPT vs. ARC-HBR	BARC ≥ 3a	0.10 (17)	0.09 (111)	0.006	0.938	−0.004	0.662
	TIMI ≥ minor	0.14 (24)	0.11 (141)	0.028	0.736	−0.015	0.001
	GUSTO ≥ mod	0.15 (17)	0.13 (165)	0.025	0.795	−0.009	0.597

NRI, net reclassification improvement; IDI, integrated discrimination improvement; other abbreviations as in Table 5.

## Data Availability

Data are the property of the authors and are available by contacting the corresponding author.
